# Association of a Combined Body Mass Index and Regional Body Fat Percentage Metric With Fragility Fracture Risk: Evidence from a Large Observational Cohort

**DOI:** 10.1002/jcsm.13808

**Published:** 2025-04-17

**Authors:** Hamzah Amin, Michelle G. Swainson, Muhammed Aqib Khan, Marwan Bukhari

**Affiliations:** ^1^ Lancaster University Lancaster UK; ^2^ Royal Lancaster Infirmary Lancaster UK

**Keywords:** adiposity, ageing population, BMI, body composition, fracture risk estimation, fragility fractures, obesity, sarcopenia, sarcopenic obesity

## Abstract

**Background:**

Evidence suggests that high body fat and low muscle mass may increase the risk of fragility fractures. However, current fracture risk models, which largely rely on body mass index (BMI), may not fully capture these compositional factors. We recommend integrating additional body composition variables into fracture risk calculators to improve accuracy. Previously, we described partial body fat percentage (PBF%), a novel measure that is routinely available and calculated as the proportion of fat at the lumbar spine and hip during DXA scans. We hypothesize that a combined BMI and PBF% approach (BMI/PBF%) could be associated with fragility fracture.

**Methods:**

Patients were referred to our DXA scanner between June 2004 and February 2024 and had combined lumbar spine and bilateral femoral scans. Patients were initially categorized by BMI (underweight, normal weight, overweight and obese) and then divided into tertiles of PBF%. Based on each patient's unique combination of BMI and PBF% tertile, they were stratified into 12 binary BMI/PBF% groups for analysis. Multivariable logistic regression models, reporting odds ratios (OR), with BMI/PBF% groups as the independent variables and fragility fractures as the dependent variable were fit, with all results adjusted for known fracture risk factors.

**Results:**

We analysed 36 235 patients (83.4% female, 16.6% male), of whom 14 342 (39.5%) reported fragility fractures. The median (IQR) age was 67.7 (57.5–75.0) years, with a BMI of 26.4 (23.3–30.2) kg/m^2^ and PBF% of 30.6% (25.5% – 35.4%). In females, those in the lowest PBF% tertile had reduced odds of fragility fractures across all BMI categories (e.g., obese low PBF%: OR 0.70, 95% CI 0.64–0.78), whereas in males, this reduction was observed only amongst overweight and obese individuals (e.g., obese low PBF%: OR 0.71, 95% CI 0.57–0.88). No association was found for patients in the middle PBF% tertile across any BMI group. In contrast, females in the highest PBF% tertile exhibited increased odds of fractures across all BMI categories except underweight (e.g., obese high PBF%: OR 1.31, 95% CI 1.22–1.42), and a similar pattern was seen in males, but limited to the overweight and obese groups (e.g., obese high PBF%: OR 1.27, 95% CI 1.04–1.55).

**Conclusion:**

High or low PBF% within BMI categories is associated with fragility fractures, challenging the traditional notion that high BMI protects against fractures. This study highlights the importance of body composition measures beyond BMI in fracture risk assessment.

## 1 Introduction

Osteoporosis (OP) is a disease characterized by low bone mass and progressive deterioration of bone tissue, resulting in increased bone fragility and a heightened, typically asymptomatic, risk of fractures [1]. The World Health Organization (WHO) defines OP as a bone mineral density (BMD) at the femoral neck that is 2.5 standard deviations below the peak bone mass of a young, healthy adult, as measured by a dual‐energy X‐ray absorptiometry (DXA) scan [2]. OP presents a major global public health challenge, affecting around 22% of women and 7% of men over the age of 50 in the United Kingdom [3]. Fragility fractures linked to OP, such as the 72 000 hip fractures reported in the United Kingdom in 2023 alone [4], are significant contributors to morbidity and mortality [5]. Furthermore, the economic burden is also substantial, costing the United Kingdom's National Health Service (NHS) an estimated £4.6 billion annually [6], a figure expected to increase due to an ageing population and a rise in risk factors associated with age‐related comorbidities, lifestyle choices and chronic conditions.

BMD is only a single component in overall fracture risk, so multiple known predictors of fractures are considered for greater accuracy in fracture calculators such as FRAX, and addressing these risk factors early may help to mitigate the clinical outcomes of OP [7]. These include but are not limited to previous fracture, steroid use, smoking status and weight status determined by body mass index (BMI) [7]. In response to an advancing evidence base, FRAX‐plus is currently in development, which includes additional predictors such as recent osteoporotic fracture, hip axis length and type 1 diabetes mellitus. However, despite growing evidence highlighting the negative effects of body fat on bone, fracture calculators have yet to incorporate body composition variables (beyond BMI) into their algorithms [8].

BMI is a widely used tool for weight classification based only on height and weight, but its overly simplistic nature does not provide an accurate representation of total or regional body composition, that is, body fat and lean mass [9], which can be acquired accurately via DXA. At a population level, it is generally accepted that higher BMI (overweight and obesity) positively correlates with BMD while lower BMI (underweight) negatively correlates with bone density; albeit, this relationship is largely dependent on BMD to be clinically meaningful [10]. However, BMI has been shown to poorly correlate with disorders characterized by muscle loss and fat gain such as sarcopenia and sarcopenic obesity [11] and is reported to ineffectively detect muscle and fat changes typically prevalent in older adults [9]. This is especially important given fragility fractures happen in older adults who are prone to compositional changes, with evidence suggesting these compositional changes may precipitate fractures [12].

Despite epidemiological evidence highlighting the positive correlation between BMI and BMD, there is a growing evidence base highlighting the negative association between excessive adiposity (which typically increases with BMI) and bone density, which could be affecting fracture risk [13, 14]. High adiposity, determined by assessment of body fat, is associated with higher circulating pro‐inflammatory cytokines [15] and adipokines [16], which can also precipitate vitamin d deficiency [13]. Furthermore, there are also mechanistic changes linked to fat infiltration and deposition within bone marrow that can compromise bone integrity [17]. Plus, the biomechanical effects of higher adiposity may alter balance and mobility, thereby heightening falls risk [18]. All these mechanisms can increase fracture risk, making adiposity a potentially important novel predictor of fragility fracture [14].

Given the emerging evidence linking adiposity and fracture risk [13, 14], it could be argued that the sole inclusion of BMI in fracture calculators is potentially placing some patients at risk in terms of preventing fractures. As a result, there is an increasing need to incorporate novel body composition measures into fracture calculators. However, performing a highly accurate total body composition DXA scan [19] on all patients is impractical due to the additional scan time and the limited clinical indications for such scans. Our research group has proposed a practical alternative: partial body fat % (PBF%) [20], derived from regional fat data obtained during routine hip and lumbar spine DXA scans and adds no extra scan time. While the majority of research focuses on body fat as a predictor of fracture in isolation [20, 21], we do not think this is the best approach given the evidence for BMI as a predictor of fracture [10]. Hence, we hypothesize that combining PBF% with BMI (BMI/PBF%) could provide a more comprehensive assessment of fracture risk by capturing both total weight over a given area (BMI) and providing an estimation of its composition. This combined metric could integrate into fracture risk calculators, working alongside BMI to enhance diagnostic utility and provide meaningful compositional insights without compromising existing predictive value.

## 2 Aims

The aim of this study is to examine the associations between a combined BMI/PBF% approach and fragility fracture in patients referred to an NHS‐based DXA clinic in the United Kingdom. Furthermore, for comparison, we will also assess the association between an isolated BMI and a stratified PBF% approach with fragility fractures.

## 3 Methods

### 3.1 Patients

Between June 2004 and February 2024, a total of 48 703 patients from Lancashire and South Cumbria in the Northwest of England were referred to the regional NHS Trust DXA clinic from both primary and secondary care. Upon arrival, patients completed questionnaires designed to gather information on OP risk factors, including self‐reported fragility fractures (defined as fractures occurring from standing height or less) and other FRAX risk factors, such as family history of fractures, smoking, current glucocorticoid therapy, rheumatoid arthritis and excessive alcohol consumption (> 3 units/day). While the specific reason for referral was noted by the technician, it was not explicitly documented in our dataset; for instance, a patient referred after experiencing a fragility fracture would simply be recorded as having had a fracture, rather than specifying the reason for referral. The technician then measured the patients' height and weight to calculate BMI before proceeding with the scan. All collected data were correlated with the patients' demographic information obtained from medical records, including age, date of birth, ethnicity, sex and postcode.

### 3.2 BMD Assessment

The majority of our patients underwent a bilateral femoral and lumbar spine scan to determine BMD, using the GE Lunar Prodigy (2004–2019) and more recently the GE Lunar iDXA (2019–present). Due to the timeframe of data collection and usage, DXA machines usually get replaced every 10 years but despite variation in the DXA machines used and GE Encore software upgrades within this study, cross calibration models have shown methodological agreement in BMD and associated T‐scores as per ISCD 2019 guidelines [22]. The left and right femoral regions were scanned separately and included the femoral neck, ward, greater trochanter and shaft, with internal rotation of the hip whereby feet were strapped to a positioner block to ensure a preferred image in line with ISCD guidelines [22]. The anterior–posterior (AP) lumbar spine scan involved legs positioned at approximately 60° on a foam block to provide a clearer image initiated at vertebrae L5, up to L1 but only the L1–L4 vertebrae were included in the BMD analysis. If there was more than 1SD variation between adjacent vertebrae, then exclusions were applied to rule out inconsistent areas of higher BMD that are often linked to degeneration, for example, osteoarthritis (ISCD Best Practice) [22].

### 3.3 Body Composition Assessment

Total body DXA is a highly accurate way of measuring total body fat percentage [19], with some population reference data available for comparison [23], though these data are limited for older adults and those with health issues. In our DXA clinic, none of the patients had undergone a total body composition scan, as this is not routine in UK clinical practice. However, for patients who underwent bilateral femoral and lumbar spine scans, the GE LUNAR system can still measure fat mass, lean mass and regional body composition based on the differential absorption of X‐rays by different tissue types at each scanned site. While these data are not displayed on bone density reports from the regional scans (lumbar spine and bilateral femoral), it is stored in the system's software and was thus available for statistical analysis.

With these available raw data, we calculated partial body fat percentage (PBF%), a measure of regional fat as a proportion of total mass in the hip and spine scan areas. The formula we used to calculate PBF% was: (Total Fat Mass (g) at the lumbar spine and bilateral femoral regions)/(Total Fat Mass (g) + Total Lean Mass (g) at said sites) × 100. It should also be noted that PBF% still requires validation against total body fat percentage (BF%), and our research group is actively working on obtaining the appropriate datasets for this comparison. Therefore, we cannot currently compare PBF% to published total BF% reference data. However, previous studies do indicate a strong correlation between regional and total body fat percentages when measured by DXA, hence the reason for the development of this novel measure [24, 25].

## 4 Data Analysis

Only patients who underwent combined lumbar spine and bilateral femoral scans were included in our analysis, excluding those with isolated scans, as we could not apply our formula to establish the PBF%. Additionally, excluding these patients minimized potential bias, providing a sample of patients that can be accurately compared to each other.

The data were initially assessed for normality through visual inspection of Q‐Q plots and histograms. Formal tests of normality, including the Shapiro–Wilk test and assessments of skewness and kurtosis, were also conducted. Normal data were presented as mean (SD) with skewed/non‐normal data presented as median (IQR). For continuous variables, the Student's *t*‐test was used. Furthermore, given the tests robustness in large samples, even in the presence of skewed data, we used the *t*‐test to analyse continuous variables [26]. Categorical variables were analysed using Pearson's chi‐squared test. Statistical significance was defined as a *p*‐value of less than 0.05.

To generate our BMI/PBF% groups, we followed a three‐step process. First, patients were stratified by their BMI category: underweight (BMI < 18.5 kg/m^2^), healthy weight (BMI 18.5–24.9 kg/m^2^), overweight (BMI 25–29.9 kg/m^2^) and obese (BMI ≥ 30 kg/m^2^). Next, patients were also divided into arbitrary tertiles based on PBF%, which was appropriate given the lack of established reference data for PBF%. Finally, patients were categorized into a combined BMI and PBF% groups, resulting in 12 distinct binary groups for analysis (e.g., an underweight patient according to BMI who is also in the tertile 1 PBF%, representing the lowest PBF%, was coded as 1, with all others coded as 0). These groups formed the basis for our statistical analysis. Prior to our analysis, frequency analysis was conducted to assess the distribution of patients across each BMI/PBF% group. A Pearson's correlation coefficient was also reported looking at the relationship between BMI and PBF%.

A multivariate logistic regression model was used to assess the association between our combined BMI/PBF% groups and any fragility fracture. Any fragility fracture was defined as hip (+ femoral) and non‐hip fractures (i.e., tibia/fibula, wrist, humerus, ankle and so forth) that occurred because of low impact trauma. Hip fractures were not analysed separately due to insufficient cases in the individual groups. The BMI/PBF% group classifications were the independent variable, while the presence of any fragility fracture was the dependent variable. Analyses were conducted separately for males and females, as females naturally have higher levels of body fat than men [27], and the peri‐/post‐menopausal hormonal changes have a role to play. All analyses were adjusted for known fracture risk factors: age, smoking, excessive alcohol consumption, glucocorticoid therapy, rheumatoid arthritis, parental history of fracture, personal history of fragility fracture and the left total femoral T‐score. A Bonferroni correction was applied to account for multiple comparisons and to reduce the risk of a type 1 error, which took the set *p* value of 0.05 and divided it by the number of groups within the analysis (i.e., 12) resulting in an adjusted significance threshold of *p* = 0.004 (i.e., 0.05/12).

A sensitivity analysis was also conducted utilizing a multivariate logistic regression, examining BMI and PBF% separately to explore whether any association was because of an individual compositional variable (BMI or PBF) or the combined approach (BMI/PBF%). BMI and PBF% were both treated as the independent variables in each analysis with fragility fractures being the dependent variable. A Bonferroni corrected *p*‐value was also applied due to multiple comparisons. The *p*‐value for our isolated PBF% analysis was set at *p* = 0.017(0.05/3 tertiles) while a *p*‐value of 0.013 (0.05/4 categories) was applied for our BMI analysis. All analyses were also adjusted for the previously mentioned confounders. Analyses were also stratified by biological sex.

All statistical analyses were conducted using Stata version 18.

## 5 Results

### 5.1 Demographics

A total of 36 235 patients who underwent combined lumbar spine and bilateral femoral scans were included in this study, comprising 30 216 females (83.4%) and 6019 males (16.6%). Amongst the cohort, 14 342 patients (39.5%) reported experiencing a fragility fracture, including 12 046 females (39.8%) and 2296 males (38.1%), with a statistically significant difference between groups (*p* = 0.013). A history of previous fracture was reported in 5.0% of females and 5.8% of males (*p* = 0.008). Additionally, approximately one in five patients had a family history of fracture, reported by 20.7% of males and 22.1% of females (*p* = 0.015).

The descriptive statistics, presented as median (IQR), demonstrated that the median age of all patients was 67.7 (57.5–75.0) years, height was 161.8 (156.5–167.5) cm, weight was 69.5 (60–81) kg and BMI was 26.4 (23.3–30.2) kg/m^2^. The median left femoral T‐score for all patients was −1.0 (−18 to −0.13). Amongst the underweight group, 891 (90%) were female compared to 98 (10%) males (*p* < 0.001). For those at a healthy BMI, 11309 (86.2%) were female, while 1804 (13.8%) were male (*p* < 0.001). Similarly, in the overweight category, 10 175 (79.9%) were female, and 2555 (20.1%) were male (*p* < 0.001). Interestingly, there was no statistically significant difference in the obesity rates between females (7841, 83.4%) and males (1562, 16.6%) based on BMI (*p* = 0.998). Pearson's correlation coefficient indicated a weak correlation between BMI and PBF% (*r* = 0.117, *p* < 0.001).

Student's *t*‐test showed that female patients were younger at the time of referral, were shorter, weighed less and had lower BMIs with higher PBF% (*p* < 0.001). Female patients also had lower left femoral T‐scores with a mean (SD) of −0.95 (1.3) versus their male counterparts, who had a mean of −0.91 (1.3) (*p* < 0.001). Pearson's chi‐squared test revealed that there were no significant gender differences in alcohol use or smoking. However, females had a significantly higher prevalence of personal and family histories of fractures, as well as reported prior fractures. In contrast, males were more likely to report glucocorticoid use than females (*p* < 0.05). No significant difference was found for rheumatoid arthritis. Further sex‐specific predictors are summarized in Table 1.

**TABLE 1:** Patient characteristics.

	Total number of patients	Female (*n* = 30 216; 83.4%)	Male (*n* = 6019; 16.6%)	*p*
Demographics				
Age (years)	36 235 (100%)	65.2 (12.8)	67.1 (13.1)	< 0.001
Height (cm)	36 235 (100%)	160.3 (7.3)	172.4 (7.4)	< 0.001
Weight (kg)	36 235 (100%)	69.6 (15.6)	81.8 (16.3)	< 0.001
Body mass index (kg/m^2^)	36 235 (100%)	27.1 (5.7)	27.4 (4.4)	< 0.001
Lifestyle factors				
Alcohol use (> 3 units per day)	2605 (7.2%)	2167 (7.2%)	438 (7.2%)	0.773
Current smoker	3885 (10.7%)	3241 (10.7%)	644 (10.7%)	0.951
Medical history				
Glucocorticoid use	4676 (12.9%)	3819 (12.6%)	857 (14.2%)	0.001
Rheumatoid arthritis	2301 (6.4%)	1893 (6.3%)	408 (6.8%)	0.136
Personal history of a fracture	1857 (5.1%)	1508 (5.0%)	349 (5.8%)	0.008
Family history of a fracture	7943 (21.9%)	6687 (22.1%)	1247 (20.7%)	0.015
Report of any fracture	1434 (39.5%)	12 046 (39.8%)	2296 (38.1%)	0.013
DXA outcomes				
Left femoral T‐score	34 895 (100%), missing = 1340 (3.7%)	−0.95 (1.3)	−0.91 (1.3)	0.03
Total fat mass at spine and hip regions (g)	36 235 (100%)	922 (221)	914 (219)	0.014
Total lean mass at spine and hip regions (g)	36 235 (100%)	2109 (220)	2117 (217)	0.007
Partial BF%	36 235 (100%)	30.4 (7.2)	30.1 (7.1)	< 0.001

*Note:* Data presented as *n* (%) for binary variable and mean (SD), median (IQR) for continuous variables. Student's *t*‐test used for continuous variables and Pearson's chi‐squared used for categorical outcomes.

Our PBF% variable had a median of 30.6% (25.5%–35.4%) across the entire population. The lowest tertile of PBF% had a median of 23.2% (20.0%–25.5%), the middle tertile had a median of 30.6% (29.0%–32.1%) and the highest tertile had a median of 37.5% (35.4%–40.3%). Female patients had a median PBF% of 30.6% (25.5%–35.4%) while male patients had a median PBF% of 30.3% (25.3%–35.1%). Additional details on the frequency of patients in each binary BMI/PBF% group are provided in Table 2.

**TABLE 2:** BMI/PBF% frequency distribution.

Patient group	BMI classification			
	Underweight (< 18.5 kg/m^2^) (*n* = 989; 2.73%)	Normal weight (18.5–24.9 kg/m^2^) (*n* = 13 113; 36.2%)	Overweight (25.0–29.9 kg/m^2^) (*n* = 12 730; 35.1%)	Obese (≥ 30 kg/m^2^) (*n* = 9403; 26.0%)
Females (*n* = 30 216; 83.4%)	*Total* 891 (90.0%)	*Total* 11 309 (86.2%)	*Total* 10 175 (79.9%)	*Total* 7841 (83.4%)
Low PBF%	355 (35.6%)	4474 (34.1%)	3100 (24.4%)	2095 (22.2%)
Moderate PBF%	261 (26.4%)	3716 (28.3%)	3655 (28.7%)	2403 (25.6%)
High PBF%	275 (27.8%)	3119 (23.8%)	3410 (26.8%)	3343 (35.6%)
Males (*n* = 6019; 16.6%)	*Total* 98 (10.0%)	*Total* 1804 (13.8%)	*Total* 2555 (20.1%)	*Total* 1562 (16.6%)
Low PBF%	33 (3.3%)	673 (5.1%)	869 (6.8%)	480 (5.1%)
Moderate PBF%	32 (3.2%)	577 (4.4%)	863 (6.7%)	561 (6.0%)
High PBF%	33 (3.3%)	554 (4.2%)	823 (6.5%)	521 (5.5%)

*Note:* All groups were statistically significant against the equivalent sex group (*p* < 0.001). For example, underweight tertile 1 male versus female and so forth.

No patients in our dataset had missing data for height, weight, age, PBF% and any of the variables we adjusted for in our logistic regression model, as was described in the methods section; however, 1340 (3.7%) of patients were missing a left femoral T‐score.

### 5.2 Fracture Association With BMI/PBF%

Female patients with low PBF% across all BMI groups had a decreased association with fragility fractures. For instance, underweight females in the lowest PBF% tertile had 31% lower odds of reporting a fracture versus the rest of the population (OR 0.69 [95% CI: 0.55, 0.88]). No significant association was observed in female patients in the moderate PBF% tertile at any BMI level. However, a significantly increased association with fragility fractures was observed in females in the highest PBF% tertile, across all BMI categories (+26% to 40% increase in odds) except underweight female patients. For example, normal weight females in the highest PBF% tertile had 40% increased odds of fracture compared to the rest of the population (OR 1.40 [95% CI: 1.29, 1.52]). These results are presented in Table 3.

**TABLE 3:** Logistic regression for fragility fractures across the combined BMI/PBF% groups (odds ratios according to BMI but stratified for PBF%).

Patient group	Females		Males	
	OR [95% CI]	*p*	OR [95% CI]	*p*
UW/low PBF%	0.69 [0.55, 0.88]	0.002	1.45 [0.69, 3.03]	0.319
UW/moderate PBF%	0.94 [0.72, 1.22]	0.644	1.30 [0.63, 2.67]	0.482
UW/high PBF%	1.26 [0.98, 1.63]	0.074	0.92 [0.41, 2.06]	0.833
NW/low PBF%	0.74 [0.68, 0.79]	< 0.001	0.94 [0.79, 1.12]	0.490
NW/moderate PBF%	1.05 [0.98, 1.12]	0.174	0.89 [0.73, 1.08]	0.228
NW/high PBF%	1.40 [1.29, 1.52]	< 0.001	1.20 [0.99, 1.46]	0.063
OW/low PBF%	0.79 [0.73, 0.86]	< 0.001	0.81 [0.69, 0.95]	0.012
OW/moderate PBF%	0.97 [0.90, 1.05]	0.484	1.05 [0.89, 1.23]	0.564
OW/high PBF%	1.26 [1.16, 1.36]	< 0.001	1.33 [1.13, 1.57]	< 0.001
Ob/low PBF%	0.70 [0.64, 0.78]	< 0.001	0.71 [0.57, 0.88]	0.002
Ob/moderate PBF%	1.01 [0.92, 1.11]	0.791	0.90 [0.74, 1.09]	0.286
Ob/high PBF%	1.31 [1.22, 1.42]	< 0.001	1.27 [1.04, 1.55]	0.021

*Note:* Green font indicates a reduced association, black font indicates no association and red font indicates an increased association.

Abbreviations: NW, normal weight; Ob, obese; OW, overweight; PBF%, partial body fat percentage; UW, underweight.

In males, a similar pattern was observed but only in the overweight and obese BMI groups. Obese and overweight males in the lowest PBF% tertile showed 19%–29% reduced odds of reporting a fragility fracture (OR 0.81, [95% CI: 0.69, 0.95]; OR 0.71 [95% 0.57, 0.88], respectively). No significant association was observed in the moderate PBF% tertile for overweight or obese males. Conversely, those in the highest PBF% tertile had a significantly increased association of fragility fractures ranging from 27% to 33% increase in odds for obese and overweight males, respectively (e.g., overweight males in the highest fat tertile had an OR of 1.33 [95% CI: 1.13, 1.57]). These results are presented in Table 3.

### 5.3 Sensitivity Analysis

Using BMI alone was not associated with fragility fractures in either male or female patients. However, when we assessed PBF% alone, a similar pattern emerged as observed in our combined BMI/PBF% analysis. In female patients, those in the lowest tertile of PBF% had 33% lower odds of reporting a fragility fracture (OR 0.67, 95% CI: 0.64–0.71), while those in the highest tertile had a 46% higher likelihood (OR 1.46, 95% CI: 1.39–1.54). Similarly, men in the lowest PBF% tertile had 22% reduced odds of reporting a fragility fracture (OR 0.78, 95% CI: 0.69–0.88), while those in the highest tertile had 37% increased odds (OR 1.37, 95% CI: 1.21–1.54). No significant association was found for patients in the middle tertile of PBF% in either gender. These results can be seen in Table 4.

**TABLE 4:** Sensitivity analysis for (a) BMI classification only and (b) PBF% only.

Patient group	Females		Males	
	OR [95% CI]	*p*	OR [95% CI]	*p*
(a)				
Underweight	0.91 [0.78, 1.06]	0.215	1.22 [0.79, 1.88]	0.374
Normal weight	1.0 [0.95, 1.05]	0.916	0.99 [0.88, 1.12]	0.907
Overweight	0.99 [0.94, 1.05]	0.769	1.06 [0.94, 1.19]	0.333
Obese	1.03 [0.97, 1.09]	0.357	0.92 [0.81, 1.05]	0.219
(b)				
Low PBF%	0.67 [0.64, 0.71]	< 0.001	0.78 [0.69, 0.88]	< 0.001
Moderate PBF%	1.01 [0.96, 1.07]	0.601	0.95 [0.84, 1.07]	0.386
High PBF%	1.46 [1.39, 1.54]	< 0.001	1.37 [1.21, 1.54]	< 0.001

*Note:* Green font indicates a reduced association, black font indicates no association and red font indicates an increased association.

## 6 Discussion

The results of our study align with expected sex differences in anthropometry and body composition, revealing that females generally have lower body weight than males while exhibiting higher levels of regional body fat, even at lower BMI levels [28]. Interestingly, though, while the difference in PBF% between males and females was statistically significant, it was a smaller difference than we would have expected. However, we know females store more fat in the gynoid (hip) region, while males tend to accumulate fat in the android (abdominal) region [27]. However, during the post‐menopausal period, females often experience a shift in body fat distribution towards the abdomen, which may account for the similar PBF% observed across sexes in this study [29]. Furthermore, since a smaller proportion of men were referred to our scanner, the male cohort may not represent the general male population but rather a subset of men at higher risk for fragility fractures.

Nonetheless, we found that across all BMI categories, female patients with low adiposity (low PBF%) demonstrated 21%–31% decreased odds of experiencing a fragility fracture, while those in the highest PBF% group faced up to a 40% increase in the odds of fracture. This finding challenges the traditional understanding that higher BMIs are protective against fragility fractures due to the associated increase in BMD [10]. Rather, our results suggest that in females, even within BMI groups, there is likely a differing level of risk depending on the amount of body fat patients have.

In male patients, the association between our combined BMI/PBF% approach and fracture incidence was significant only within the overweight and obese BMI groups. In these groups, low PBF% was linked to up to a 29% reduction in fragility fracture odds, while high PBF% was associated with up to a 33% increase in fracture odds. This further supports the notion that fracture risk can vary even within BMI categories and is influenced by body fat percentage. However, our results suggest that sex‐based differences warrant further investigation, as our findings were only observed in overweight and obese males, while in females, the association was seen across most BMI groups. It is, however, plausible that overweight and obese males with high PBF% have proportionally lower lean mass and muscle quality due to the increased anti‐androgenic effects of excessive adiposity, which may contribute to increased fracture risk [30]. In contrast, in patients with lower BMIs, the actual fat mass may not be sufficient to exert the same anti‐androgenic effects, suggesting that muscle strength could be better preserved at lower BMIs, regardless of PBF% [30]. This may explain why elevated PBF% in males does not necessarily increase fracture odds in underweight and normal‐weight BMI groups. However, rather than drawing definitive conclusions from this data, we recommend further studies with larger male cohorts to better understand potential sex differences in fracture risk when utilizing our approach.

Our sensitivity analysis demonstrated that distributional PBF% tertiles also had a distinct association with fragility fractures, similar to our combined BMI/PBF% approach, with high PBF% associated with increased odds and low PBF% associated with decreased odds across male and female patients. Interestingly, we found no association between an isolated BMI approach and fragility fractures. Based on previous literature, we would have expected to observe a decreased odds of fracture in patients with higher BMI; however, this trend was not reflected in our dataset [10]. This finding underscores the necessity for routine body composition measurements to better predict fracture risk, as BMI alone may overlook at‐risk individuals within each BMI category. While our analysis in Table 4 indicates that PBF% predominantly drives the observed association in our combined BMI/PBF% approach, the differing relationships observed between males and females suggest that a combined approach may be better suited to detecting potential sex‐specific variations, as previously described, though further research is needed to confirm this.

Even though we have not measured muscle mass directly, we do believe the reciprocal relationship between fat and lean mass may also provide an indirect framework for considering muscle in measures primarily using adiposity, including our BMI/PBF% measurement [31]. Individuals with low adiposity are often assumed to have higher muscle and lean mass, which may provide protection against fractures through enhanced stability and increased bone mineral density (BMD) [32]. This is reflected in the decreased odds of fragility fractures observed in females with low PBF% across all BMI categories, as well as in overweight and obese males with low PBF%. In contrast, individuals with high adiposity may have reduced muscle mass, with any remaining muscle potentially compromised by fat infiltration, leading to diminished muscle quality and quantity [33]. This could result in instability and falls [18], thereby increasing fracture risk. In our study, we observed an increased association with fragility fracture in the majority of high PBF% groups in females and in overweight and obese males. These dynamics may contribute to the association observed between our combined BMI/PBF% approach and fragility fractures. Similarly, this could also explain the association seen in our isolated PBF% approach.

Our results are particularly significant considering the growing evidence linking sarcopenia, sarcopenic obesity, and more recently, osteoporotic‐sarcopenic obesity (OSO) to fracture risk [34, 35]. There is considerable value in identifying a clinically useful and straightforward tool that offers insights into both fat and muscle composition. Previous research has shown that muscle mass assessment has been inconsistent in its prediction of fragility fracture [36, 37], so potentially it is the body fat component that is most closely related to fracture. Notably, research by Kelly et al [38] on OSO suggests that assessing regional fat, particularly visceral and abdominal adiposity, is more relevant to health outcomes than overall body weight. This further supports the use of PBF% as a practical measure of regional fat that can be obtained without the need for a total body scan in addition to routine bone scans. However, validation with visceral fat would be needed to further support its use in relation to OSO diagnosis.

We believe our calculated BMI/PBF% is a practical variable that could enhance fracture risk calculators by capturing key compositional factors. However, further research is needed to expand on our approach. Future studies should validate PBF% against gold‐standard assessments, such as DXA‐derived visceral fat [39], and explore its relationship with DXA‐derived total body fat [25]. It would also be valuable to establish thresholds for low, moderate and high PBF% in reference populations and assess their predictive power for fractures, both independently and in combination with BMI. Additionally, investigating hormonal profiles, such as anti‐androgenic responses in men [30] and the hormonal changes associated with menopause [40], along with their correlation to body composition and fracture risk, would provide valuable insights into gender differences in fracture risk. We encourage replication of our approach across diverse male populations, ethnicities and age groups. If confirmed, PBF% could be incorporated as an additional risk factor for fracture prediction in future FRAX tool updates.

## 7 Strengths and Limitations

The main strength of our study is the relatively large number of patients included, all of whom had the gold standard assessment for BMD (DXA). We also adjusted for known predictors of fractures, which strengthens our results. While total body composition scans provide detailed insights, their clinical utility is limited by the additional time and software required. Hence, we believe our PBF% method is a strength of our study, as it uses routinely collected data, requires no extra effort and remains associated with fragility fractures even after adjusting for fracture predictors.

Our study has several limitations. First, the cross‐sectional design restricts our ability to observe changes over time, particularly in assessing whether PBF% or BMI/PBF% predicts future fractures, making longitudinal follow‐up essential. This would help determine whether compositional changes precede fractures or if fractures themselves trigger shifts in body composition. Additionally, it remains unclear whether an initial fracture induces compositional changes that subsequently increase the risk of future fractures.

Additionally, the homogeneity of our predominantly Caucasian, at‐risk population limits generalizability to the broader UK population, though it accurately reflects the demographics of North Lancashire and South Cumbria. Furthermore, since these patients were referred, they likely have more comorbidities than the general public, which may further limit the generalizability of our findings.

## 8 Conclusion

To our knowledge, our study is one of the first to demonstrate that high or low PBF% within BMI categories is associated with fragility fractures, challenging the traditional notion that high BMI protects against fractures. Specifically, we found that higher PBF% increases the odds of fragility fractures in females across most BMI categories (excluding underweight) and in males within the overweight and obese categories. Conversely, lower PBF% was linked to decreased odds of fragility fractures in females across all BMI groups and in overweight and obese males. However, further research with larger male datasets is needed to confirm potential sex differences. Our study contributes to the growing body of literature highlighting the importance of body composition in fracture risk assessment. It also provides practitioners with a practical, resource‐efficient method for evaluating regional body composition and demonstrates how such measurements can be integrated into fracture calculators, utilizing routinely collected data without increasing scan time or resource demands. Further research is needed to validate our findings in longitudinal cohorts.

## Ethics Statement

Full ethical approval for pseudonymized data extraction in the absence of informed consent was obtained from the regional NHS Research Ethics Committee Northwest Preston (project number 14/NW/1136).

## Conflicts of Interest

H.A., M.G.S. and M.A.K. declare no conflicts of interest.

M.B. has the following disclosures: M.B. has been sponsored to attend regional, national and international meetings by UCB Celltech, Roche/Chugai, Pfizer, Abbvie, Merck, Mennarini, Janssen, Bristol‐Myers Squib, Novartis and Eli Lilly. He has received honoraria for speaking and attended advisory boards with Bristol‐Myers Squib, UCB Celltech, Roche/Chugai, Pfizer, Abbvie, Merck, Mennarini, Sanofi‐Aventis, Eli Lilly, Janssen, Amgen, Novartis and Gilead. He has received honoraria from educational groups Revalidaid and TREG consultants.
